# Fetal MRI radiomics: non-invasive and reproducible quantification of human lung maturity

**DOI:** 10.1007/s00330-022-09367-1

**Published:** 2023-01-06

**Authors:** Florian Prayer, Martin L. Watzenböck, Benedikt H. Heidinger, Julian Rainer, Victor Schmidbauer, Helmut Prosch, Barbara Ulm, Erika Rubesova, Daniela Prayer, Gregor Kasprian

**Affiliations:** 1 Department of Biomedical Imaging and Image-Guided Therapy, Medical University of Vienna, Spitalgasse 23, 1090 Vienna, Austria; 2 Department of Obstetrics and Gynecology, Medical University of Vienna, Spitalgasse 23, 1090 Vienna, Vienna Austria; 3 Department of Pediatric Radiology, Lucile Packard Children’s Hospital at Stanford, Stanford University, 725 Welch Road, Stanford, CA 94305 USA; 4 Imaging Bellaria, Bellariastrasse 3, 1010 Vienna, Austria

**Keywords:** Fetal imaging, Magnetic resonance imaging, Lung, Reproducibility of results

## Abstract

**Objectives:**

To assess the reproducibility of radiomics features extracted from the developing lung in repeated in-vivo fetal MRI acquisitions.

**Methods:**

In-vivo MRI (1.5 Tesla) scans of 30 fetuses, each including two axial and one coronal T2-weighted sequences of the whole lung with all other acquisition parameters kept constant, were retrospectively identified. Manual segmentation of the lungs was performed using ITK-Snap. One hundred radiomics features were extracted from fetal lung MRI data using Pyradiomics, resulting in 90 datasets. Intra-class correlation coefficients (ICC) of radiomics features were calculated between baseline and repeat axial acquisitions and between baseline axial and coronal acquisitions.

**Results:**

MRI data of 30 fetuses (12 [40%] females, 18 [60%] males) at a median gestational age of 24 + 5 gestational weeks plus days (GW) (interquartile range [IQR] 3 + 3 GW, range 21 + 1 to 32 + 6 GW) were included. Median ICC of radiomics features between baseline and repeat axial MR acquisitions was 0.92 (IQR 0.13, range 0.33 to 1), with 60 features exhibiting excellent (ICC > 0.9), 27 good (> 0.75–0.9), twelve moderate (0.5–0.75), and one poor (ICC < 0.5) reproducibility. Median ICC of radiomics features between baseline axial and coronal MR acquisitions was 0.79 (IQR 0.15, range 0.2 to 1), with 20 features exhibiting excellent, 47 good, 29 moderate, and four poor reproducibility.

**Conclusion:**

Standardized in-vivo fetal MRI allows reproducible extraction of lung radiomics features. In the future, radiomics analysis may improve diagnostic and prognostic yield of fetal MRI in normal and pathologic lung development.

**Key Points:**

*• Non-invasive fetal MRI acquired using a standardized protocol allows reproducible extraction of radiomics features from the developing lung for objective tissue characterization.*

*• Alteration of imaging plane between fetal MRI acquisitions has a negative impact on lung radiomics feature reproducibility.*

*• Fetal MRI radiomics features reflecting the microstructure and shape of the fetal lung could complement observed-to-expected lung volume in the prediction of postnatal outcome and optimal treatment of fetuses with abnormal lung development in the future.*

**Supplementary Information:**

The online version contains supplementary material available at 10.1007/s00330-022-09367-1.

## Introduction

Radiomics describes an image analysis process, where a large number of quantitative features are extracted from image data using predefined statistical operations [1]. The aim of radiomics is to identify visually imperceptible image features that characterize a specific tissue or predict a certain outcome, thereby maximizing the extraction of potentially useful information from medical images [2]. While this concept has thus far primarily been applied to oncologic imaging to improve outcome prediction [3, 4], recently it is increasingly used in non-oncologic imaging, including in-vivo fetal imaging of the developing lung [5].

Fetal MRI plays a major role in the assessment of cases with sonographically suspected abnormalities of lung development [6]. It helps guide the managing team and parents in optimizing peri- and postnatal treatment planning, which may include intubation and/or extracorporeal membrane oxygenation [7]. While conventional visual assessment of fetal MRI requires considerable expertise and years of experience, fetal MRI data is well-suited for radiomics analysis owing to the fact that image acquisition is performed in a standardized fashion according to internationally accepted ISUOG Practice Guidelines [8]. Objective quantitative image analysis using radiomics in addition to subjective visual interpretation of fetal MRI findings has the potential to improve tissue characterization, and accuracy of outcome prediction in cases with abnormal lung development. In the future, fetal MRI radiomics features correlating with postnatal clinical parameters, such as the need for mechanical ventilation or extra-corporeal membrane oxygenation, could help guide clinicians and parents with regard to optimal postnatal treatment planning. Additionally, fetal MRI radiomics may facilitate access to state-of-the art diagnostics in places with limited resources or expertise.

However, some radiomics features have been shown to be affected by alterations in image acquisition parameters, and repeated acquisitions [9, 10]. Preceding its targeted application in the imaging assessment of developmental lung diseases (for instance pulmonary hypoplasia due to premature rupture of membranes, congenital diaphragmatic hernia, etc.), radiomics feature reproducibility analysis is an essential prerequisite [2]. Currently, there is a lack of evidence regarding the robustness of quantitative radiomics features, particularly of the lung, extracted from fetal MRI data against repeated image acquisition. Therefore, this study was performed to assess the reproducibility of one hundred first- and second-order radiomics features extracted from the fetal lung in repeated in vivo MRI acquisitions using the open-source package Pyradiomics [11], which has been widely used in lung imaging and beyond.

## Methods

This retrospective study was approved by the institutional review board of the Medical University of Vienna, and the need for informed consent was waived.

### Patients

In accordance with previous test-retest studies assessing the robustness of radiomics features in repeated MRI examinations [10, 12, 13], thirty cases were retrospectively included. Fetuses with normal and fetuses with pathologic lung development were included in this radiomics feature reproducibility study. The hospital image database containing clinically indicated routine fetal MRI scans was searched for examinations that included repeated axial and coronal T2-weigthed sequences of the fetal lung between January 2016 and February 2022. Cases were excluded for lack of ultrasound-based gestational age, presence of MR artefacts, such as fetal or maternal motion, incomplete representation of the lungs, or if the lung was visible on less than 5 images in any image stack. Gestational age calculated based on ultrasound examination was recorded and is given in weeks plus days post menstruation.

### Fetal MRI

All fetal MRI data were acquired for clinical routine purposes using one 1.5-T scanner (Ingenia, Philips Healthcare) and a body coil. Indications for fetal MRI consisted of sonographically suspected organ malformations, such as ventriculomegaly, macrocephaly, or focal lung lesion, and sonographically unclear situations due to oligo- or anhydramnios. Repeated T2-weighted MRI acquisitions of the fetal lungs were routinely obtained in order to ensure artifact-free image data for lung volumetry, and—if found to be present—to allow confirmation of pulmonary hypoplasia based on the second acquisition, according to the clinical referral. In each case, two axial and one coronal acquisition of a standardized T2-weighted sequence were acquired using the following parameters: field of view 200 to 300 mm, slice thickness 3 to 4 mm (thinner slices used in early gestation), gap 0.3 to 0.4 mm, 256 x 256 matrix, shortest repetition time (7536.2 to 31,575 msec), echo time 100 ms, and flip angle 90°. No sedation or contrast was administered, and specific absorption rate levels exceeded 2W/kg bodyweight in none of the fetal MRI scans. Acquisition times of each sequence, times between the start points of baseline and repeat axial, and baseline axial and coronal MR acquisitions were recorded. In eight fetuses, two coronal T2-weighted acquisitions were available, and a subgroup analysis to assess radiomics feature reproducibility in repeated coronal acquisitions was performed.

### Radiomics

**Fig. 1:**
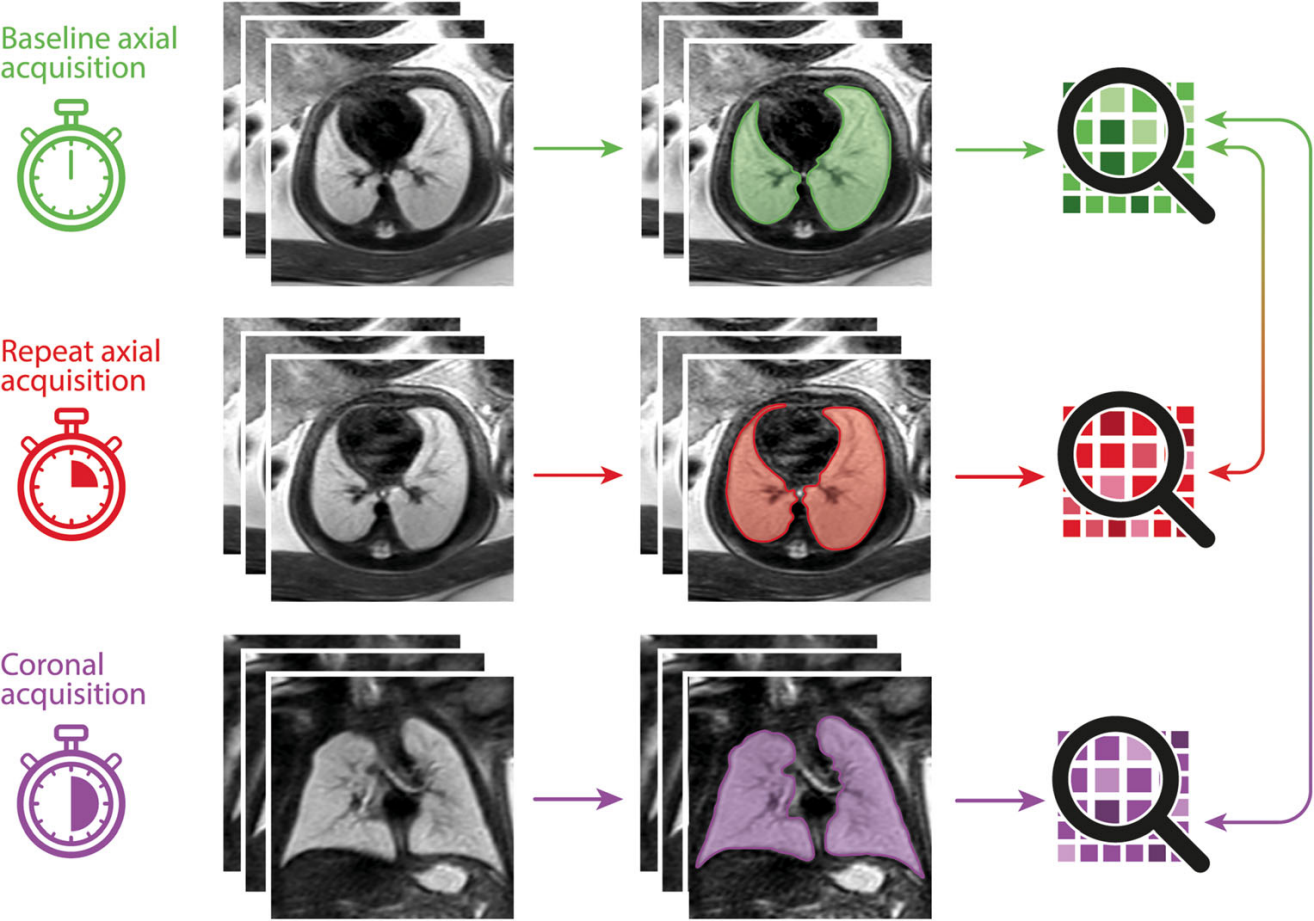
Graphical summary illustrating the segmentation and feature extraction process: A baseline axial T2-weighted sequence of the fetal lungs was acquired, and the lung manually segmented on all images. Three-dimensional lung masks were used to extract radiomics features from fetal MRI image data (top row, shown in green). This process was in a repeat axial acquisition (middle row, shown in red), and a coronal acquisition (bottom row, shown in purple). Lung radiomics features extracted from the baseline axial acquisition (shown in green) were compared to features extracted from the repeat axial (shown in red) and coronal (shown in purple) acquisitions to assess their reproducibility

Following image data anonymization and export from the institute’s PACS (Dedalus HealthCare), manual segmentation of the lungs was performed on all 30 baseline axial, 30 repeat axial, and 30 coronal image stacks using open-source software ITK-Snap [14]. Lung segmentation included abnormal lung areas in cases with pathologic lung development and was performed by one radiologist with five years of experience in fetal MRI (F.P.). Lung segmentation masks and MRI images were exported as nifti-files, and radiomics features were extracted using the open-source Python package pyradiomics running under Python 3.7.1 [11]. Image normalization was enabled by setting the *normalize* parameter to 'true' and the *normalizeScale* parameter to 100. To prevent grey values below the mean from becoming negative when normalising, the *voxelArrayShift* parameter was set to 300 (3 SDs x 100), so that only outlier values > 3 SDs below the mean would remain negative. Images were discretized, as needed, with the *binWidth* parameter set to 5. For resampling, the *interpolator* was defined as ‘sitkBSpline’, and the *resampledPixelSpacing* parameter was set to '[2,2,2]'. Radiomics features from the following feature classes were included: First Order (*n* = 18), 3D Shape (*n* = 14), Grey Level Co-occurrence Matrix (GLCM, *n* = 22), Grey Level Size Zone Matrix (GLSZM, *n* = 16), Grey Level Run Length Matrix (GLRLM, *n* = 16), and Grey Level Dependence Matrix (GLDM, *n* = 14). A complete list of radiomics features is given in the supplementary material. In total, ninety radiomics feature value sets were extracted from 30 baseline axial, 30 repeat axial, and 30 coronal MR acquisitions. Figure 1 shows the radiomics feature extraction process.

### Statistical evaluation

Statistical analysis was conducted using R version 4.0.5 (R Core Team). Intra-class correlation coefficients (ICC) were calculated to assess radiomics feature reproducibility extracted from repeated fetal MR acquisitions using the psych R package (version 2.1.9). The two-way mixed effects model (ICC3) and single-rater unit were applied. This was performed, firstly, between baseline and repeat axial fetal MRI acquisitions, and, secondly, between baseline and repeat axial and coronal acquisition. Radiomics feature reproducibility was considered excellent for ICCs > 0.9, good for > 0.75 to 0.9, moderate for 0.5 to 0.75, and poor for < 0.5 [15].

## Results

**Fig. 2:**
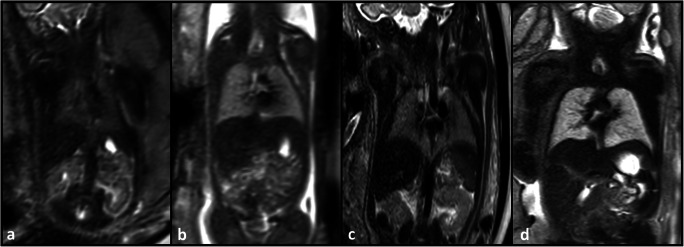
Coronal T2-weighted fetal MR images (**a**–**d**) of two fetuses with premature rupture of membranes and subsequent anhydramnios and pulmonary hypoplasia (**a**, **c**) at gestation weeks 23 (**a**) and 32 (**c**), and of two fetuses with normal lung development (**b**, **d**) at gestation weeks 23 (**b**) and 32 (**d**). Fetuses with premature rupture of membranes, anhydramnios and pulmonary hypoplasia exhibit hypointense lung tissue (**a**, **c**) compared to the lung tissue of normal controls at the same gestational age (**b**, **d**). Radiomics analysis can quantify deviations in shape and microstructural tissue qualities and may in the future complement lung volume to improve prenatal assessment of lung development

MRI data of 12 (40%) female and 18 (60%) male fetuses, acquired at a median gestational age of 24 + 5 gestation weeks (GW) (IQR 3 + 3 GW, range 21 + 1 to 32 + 6 GW) were included in this study. Pulmonary development was unremarkable in 21 of 30 (70%), eight of 30 (26.7%%) had pulmonary hypoplasia due to oligo- or anhydramnios, and one of 30 (3.3%) had a focal lung lesion (see the Supplementary Material for a complete list of fetal pathologies). Figure 2 provides an example of abnormal lung development affecting the microstructure of the fetal lung. Mean maternal age was 29 years (IQR 7.4 years, range 17.7 to 42.3 years). Median acquisition times of the initial axial, repeat axial, and coronal T2-weighted sequences were 1.3 min (IQR 0.7 min, range 0.7 to 2.5 min), 1.4 min (IQR 0.8 min, range 0.6 to 2.3 min), and 1.3 min (IQR 0.3 min, range 0.5 to 2.8 min), respectively. The median time intervals between baseline and repeat axial, and baseline axial and coronal acquisitions were 4 min (IQR 5.8 min, range 0.7 to 24.2 min) and 4.8 min (IQR 4.5 min range 0.9 to 22.6 min), respectively.

**Fig. 3:**
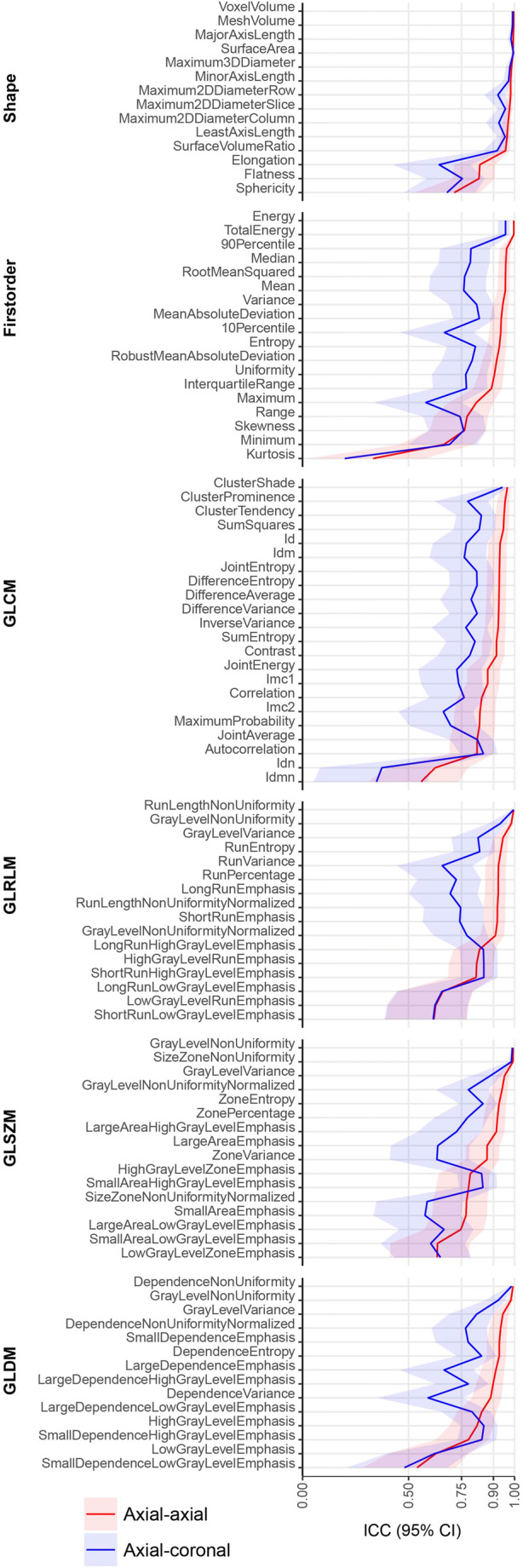
Intra-class correlation coefficients for all 100 extracted radiomics features between baseline and repeat axial acquisitions (red line), and between baseline axial and coronal acquisitions (blue line). CI confidence interval, GLCM Grey Level Co-occurrence Matrix, GLDM Grey Level Dependence Matrix, GLRLM Grey Level Run Length Matrix, GLSZM Grey Level Size Zone Matrix, ICC Intra-class correlation coefficient

**Fig. 4:**
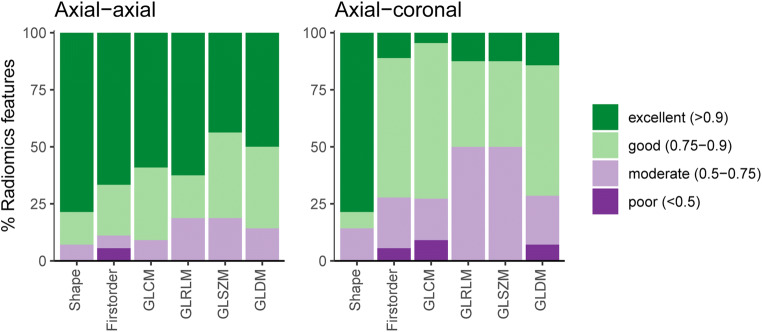
Percentages of features showing excellent (intra-class correlation coefficient > 0.9), good (0.75–0.9), moderate (0.5–0.75), and poor ( < 0.5) reproducibility between baseline and repeat axial acquisitions (left), and between baseline axial and coronal acquisitions (right). Features are grouped according to classes, and percentages are illustrated as barplots. GLCM Grey Level Co-occurrence Matrix, GLDM Grey Level Dependence Matrix, GLRLM Grey Level Run Length Matrix, GLSZM Grey Level Size Zone Matrix

For radiomics features extracted from baseline and repeat axial T2-weighted sequences, the median ICC was 0.92 (IQR 0.13, range 0.33 to 1) (Fig. 3). Reproducibility of the 100 analyzed radiomics features in baseline and repeat axial MR acquisitions was excellent in 60 (60%), good in 27 (27%), moderate in 12 (12%), and poor in 1 (1%) (Fig. 4).

For radiomics features extracted from baseline axial and coronal T2-weighted sequences, the median ICC was 0.79 (IQR 0.15, range 0.2 to 1) (Fig. 3). Radiomics feature reproducibility between baseline axial and coronal MR acquisition was found to be excellent in 20 (20%), good in 47 (47%), moderate in 29 (29%), and poor in 4 (4%) (Fig. 4). The ten best reproducible radiomics features between baseline and repeat axial and baseline axial and coronal acquisitions are given in Table 1. Table 2 shows radiomics features with excellent reproducibility between both baseline and repeat axial, and baseline axial and coronal acquisitions.

**Table 1:** Top ten reproducible radiomics features

Top 10	Feature name	Feature class	ICC
Axial vs. axial	Energy	First order	0.996
	TotalEnergy	First order	0.996
	VoxelVolume	Shape	0.996
	MeshVolume	Shape	0.996
	RunLengthNonUniformity	GLRLM	0.996
	MajorAxisLength	Shape	0.994
	GrayLevelNonUniformity	GLSZM	0.993
	DependenceNonUniformity	GLDM	0.993
	SurfaceArea	Shape	0.993
	SizeZoneNonUniformity	GLSZM	0.992
Axial vs. coronal	RunLengthNonUniformity	GLRLM	0.995
	SurfaceArea	Shape	0.995
	VoxelVolume	Shape	0.991
	MeshVolume	Shape	0.990
	GrayLevelNonUniformity	GLSZM	0.990
	DependenceNonUniformity	GLDM	0.986
	SizeZoneNonUniformity	GLSZM	0.985
	MajorAxisLength	Shape	0.983
	Maximum3DDiameter	Shape	0.978
	MinorAxisLength	Shape	0.972

Top ten reproducible fetal MRI lung radiomics features according to intra-class correlation coefficients between baseline and repeat axial, and between baseline axial and coronal acquisitions. *GLCM* Grey Level Co-occurrence Matrix, *GLDM* Grey Level Dependence Matrix, *GLRLM* Grey Level Run Length Matrix, *GLSZM* Grey Level Size Zone Matrix

**Table 2:** Radiomics features with excellent reproducibility

Feature class	Feature name	ICC axial vs. axial	ICC axial vs. coronal
Shape	VoxelVolume	0.996	0.991
	MeshVolume	0.996	0.990
	MajorAxisLength	0.994	0.983
	SurfaceArea	0.993	0.995
	Maximum3DDiameter	0.985	0.978
	MinorAxisLength	0.981	0.972
	Maximum2DDiameterRow	0.981	0.921
	Maximum2DDiameterSlice	0.976	0.956
	Maximum2DDiameterColumn	0.969	0.926
	LeastAxisLength	0.964	0.955
	SurfaceVolumeRatio	0.957	0.918
First order	Energy	0.996	0.957
	TotalEnergy	0.996	0.957
GLCM	ClusterShade	0.966	0.943
GLRLM	RunLengthNonUniformity	0.996	0.995
	GrayLevelNonUniformity	0.984	0.932
GLSZM	GrayLevelNonUniformity	0.993	0.990
	SizeZoneNonUniformity	0.992	0.985
GLDM	DependenceNonUniformity	0.993	0.986
	GrayLevelNonUniformity	0.983	0.923

Fetal MRI lung radiomics features with excellent reproducibility (intra-class correlation coefficient > 0.9) both between baseline and repeat axial acquisitions and between baseline axial and coronal acquisitions. *GLCM* Grey Level Co-occurrence Matrix, *GLDM* Grey Level Dependence Matrix, *GLRLM* Grey Level Run Length Matrix, *GLSZM* Grey Level Size Zone Matrix, *ICC* intra-class correlation coefficient

The median segmented lung volumes (shape feature MeshVolume) were 24.97 mL (IQR 17.01 mL) for baseline axial, 24.15 mL (IQR 18.23 mL) for repeat axial, and 23.95 mL (IQR 19.49 mL) for the coronal acquisitions. MeshVolume showed a high reproducibility between baseline and repeat axial (ICC 0.996), and baseline axial and coronal (ICC 0.99) MRI acquisitions. A complete list of radiomics feature ICCs is provided in the Supplementary Material.

The subgroup analysis of lung radiomics feature reproducibility in repeated coronal T2-weighted acquisitions in 8 of 30 (26.7%) fetuses showed similar feature ICCs compared to repeated axial acquisitions (see Supplementary Figures 1 and 2).

## Discussion

In this study, radiomics feature reproducibility in repeatedly acquired in-vivo fetal MRI was assessed. Excellent reproducibility of a majority of radiomics features extracted from the developing lungs in a transparent process was demonstrated if image acquisition parameters remained constant. Alteration of imaging planes in repeated acquisitions had a negative impact on radiomics feature reproducibility, decreasing the pool of highly reproducible features. These results validate the use of radiomics, i.e. visually not appreciable yet potentially clinically relevant quantitative image features, in fetal MRI of the lung. Combining visual and quantitative radiomics-based assessment of the developing lung has the potential to advance fetal MRI by increasing the amount of relevant information that can be extracted from routinely acquired image data [16, 17].

Fetal MRI is an elegant tool for the evaluation of fetal lung development, as—using T2-weighted sequences—it provides insights into the microstructural expansion of fetal future airspaces reflected by an increase in lung signal intensity [18]. However, the possibility for diagnostic exploitation of this phenomenon remains limited, as lung signal intensities show a wide variation at a given gestational stage, and are influenced by a variety of technical factors, such as field strength, fetal and coil position, number and position of coil elements, B = 0 inhomogeneities, maternal habitus, and others. Due to this lack of robustness, the evaluation of microstructural lung tissue properties using signal intensity quantification by MRI was never systematically introduced into clinical practice. Despite its initial scientific assessment in 2004 by Osada et al [19], and vast experiences by different groups with variable success [7, 18, 20–27] (see Table 3), this approach never reached the capabilities of MR-based fetal lung volumetry and the parameter of observed-to expected lung volumes in the detection and prognostic assessment of pulmonary hypoplasia [28]. However, complementing lung size and growth by tissue-specific markers reflecting lung maturity is still a plausible and promising line of research to improve diagnostic specificity and prognostic accuracy in these cases.

**Table 3:** Previous studies assessing lung signal intensity ratios

Group	Quantitative parameter	Research goal	Cases	Success
Keller et al [20]	Lung-to-liver, lung-to-amniotic fluid, lung-to-muscle SIRs	Distinguish between normal and impaired lung development	*n* = 35	No
Brewerton et al [21]	Lung-to-liver SIR	Detect pulmonary hypoplasia after 25 gestational weeks	*n* = 141	Yes
Oka et al [22]	Lung-to-liver SIR	Predict postnatal respiratory insufficiency	*n* = 110	Yes
Moshiri et al [23] , Ogawa et al [24]	Lung-to-liver SIR	Demonstrate association with gestational age	*n* = 82, *n* = 81	Yes
Mills et al [25]	Lung-to-liver, lung-to-spleen, lung-to-muscle SIRs	Demonstrate association with gestational age	*n* = 335	Yes
Matsushita et al [26], Balassy et al [18]	Lung-to-liver SIR	Predict survival in congenital diaphragmatic hernia cases	*n* = 15, *n* = 25	No
Yamoto et al [27], Dutemeyer et al [7]	Lung-to-liver SIR	Predict survival in congenital diaphragmatic hernia cases	*n* = 33, *n* = 125	Yes

Previous fetal MRI studies investigating the use of signal intensity ratios for quantitative assessment of lung development. SIR signal intensity ratio

Fetal MRI provides standardizable three-dimensional image data acquisition of the lung and different tissue contrasts (e.g. T1, T2, diffusion-weighted images, echoplanar imaging, etc.), making it well-suited for radiomics analysis. Furthermore, fetal MRI is highly useful even in cases with oligo- or anhydramnios where ultrasound assessment is difficult but lung changes can be expected. For these reasons, fetal MRI radiomics has the potential to provide useful quantitative features for tissue characterization, as previously shown in the fetal brain: Sanz-Cortés et al extracted texture features from brain MRI in GW 37 of fetuses with adequate versus delayed growth and developed a regression model to distinguish these groups with an accuracy of more than 90% [29]. The same group went on to show that fetal MRI brain texture features could identify small for gestational age fetuses with impaired neonatal behaviour [30]. Radiomics analysis of the fetal lung based on ultrasound was first explored in 1985 by Cayea et al, who failed to show an association between texture features and tissue maturity [31]. Since then, Palacio’s group showed non-invasive prediction of lung maturity, and neonatal respiratory distress based on ultrasound radiomics analysis delivers comparable accuracy to amniocentesis [32–34].

In order to ensure the safe and meaningful application of radiomics, radiomics features must be robust against technical parameters in order to reflect (patho-) physiological tissue characteristics rather than factors associated with image acquisition. Therefore, a radiomics feature reproducibility analysis, such as a test-retest experiment is required by the Radiomics Quality Score, which was proposed by Lambin et al as a benchmark for high-quality radiomics research [2]. Despite the critical importance of radiomics feature reproducibility analyses, there is a paucity of evidence in fetal imaging. One study by Perez-Moreno et al found that gray-level co-occurrence matrix, local binary patterns, and rotation-invariant local phase quantization delivered reproducible texture features from different lung regions in ultrasound images [35]. However, fetal ultrasound-based lung radiomics analysis has thus far been performed based on two-dimensional image data at the level of the four-chamber view, in lung tissue that is representative of the whole lung according to the examiner’s subjective impression. This approach is prone to introduce variation due to fetal heart positioning, fetal body position, imaging depth, and sonographer experience, among other factors. Furthermore, fetal ultrasound is impaired by oligo- or anhydramnios, where lung pathology is common.

This study demonstrates the high reproducibility of a majority of radiomics features extracted from three-dimensional fetal MRI data of the entire fetal lung. Critically, the presented results highlight the large proportion of radiomics feature with excellent reproducibility in repeated acquisitions is larger if acquisition parameters including imaging plane are kept constant. In the case of imaging plane alteration, particularly the number of second-order features (GLCM, GLDM, GLRLM, and GLSZM), which are likely to reflect relevant but visually not perceptible tissue characteristics, is reduced (37 vs 7 second-order radiomics features with ICC > 0.9). Therefore, the presented data indicate that—if used in a consistent fashion in a consistent (preferentially axial) imaging plane—radiomics prove to be the first robust approach to gain insights into lung maturity and its microstructural tissue properties by non-invasive MR imaging. Specifically, radiomics-based analysis of fetal lung development in fetal MRI may identify reproducible predictors of postnatal respiratory outcome, such as the need for mechanical ventilation or extra-corporeal membrane oxygenation. Thus, fetal MRI lung radiomics may complement observed-to-expected fetal lung volume in guiding clinicians and parents with regard to optimal postnatal management in the future. The demonstrated reproducibility of fetal MRI lung radiomics features and the potential impact of this technique on postnatal outcome prediction encourage the systematic application of fetal MRI radiomics in the assessment of developmental pathologies of the fetal lung.

There are several limitations to this study. First, the included sample size of thirty cases is small but comparable to previous works investigating radiomics feature reproducibility in test-retest studies [10, 12, 13]. Second, this study was performed using the same 1.5-T scanner at a single center, limiting the generalizability of the presented findings. However, in most fetal imaging centers, one scanner is reserved and optimized for fetal MRI. Third, the impact of motion artefacts on radiomics feature reproducibility was not assessed as respective cases were excluded. Future studies to evaluate the impact of motion, including post-processing techniques for artifact reduction, on radiomics feature reproducibility are needed. Fourth, this study only assessed radiomics features extracted from T2-weighted images - which have so far been shown to be the most promising in the assessment of fetal lung growth. Finally, human segmentation of fetal lungs may have introduced variability. However, the presented results show high ICCs between segmented lung volumes for repeated acquisitions, which is likely due to the clarity of lung visualization on fetal MRI. Previous studies confirm lung segmentation can be performed reliably, even in severe pulmonary hypoplasia [36, 37].

In conclusion, this study demonstrates a high reproducibility of a majority of radiomics features extracted from the fetal lung in repeated standardized MR acquisitions using a transparent process. This provides validation for the safe and meaningful use of radiomics in fetal MRI of normal and pathologic lung development. Caution is warranted if different imaging planes are used, as the pool of highly reproducible potential image biomarkers decreases. Thus, provided standardized (preferentially axial) image acquisition is performed, fetal MRI radiomics has the potential to increase the diagnostic and prognostic yield of fetal MRI of the developing lung in the future.

## Supplementary information

ESM 1 (DOCX 3663 kb)

ESM 2 (XLSX 14 kb)

ESM 3 (XLSX 9 kb)

## Abbreviations

GLCM: Grey Level Co-occurrence Matrix
GLDM: Grey Level Dependence Matrix
GLRLM: Grey Level Run Length Matrix
GLSZM: Grey Level Size Zone Matrix
GW: Gestational week
ICC: Intra-class correlations coefficient
IQR: Inter-quartile range
MRI: Magnetic resonance imaging

## Declarations

### Guarantor

The scientific guarantor of this publication is Gregor Kasprian, MD.

### Statistics and biometry

One of the authors (Martin Watzenboeck, MD) has significant statistical expertise. No complex statistical methods were necessary for this paper.

### Methodology

• retrospective

• experimental

• performed at one institution
