# Supplementary figures and images for: Fetal MRI radiomics: non-invasive and reproducible quantification of human lung maturity
Source: Eur Radiol. 2023 Jan 6;33(6):4205–13. doi: 10.1007/s00330-022-09367-1 (PMC10182107; doi:10.1007/s00330-022-09367-1)

# Supplementary figures

Supplementary figure 1


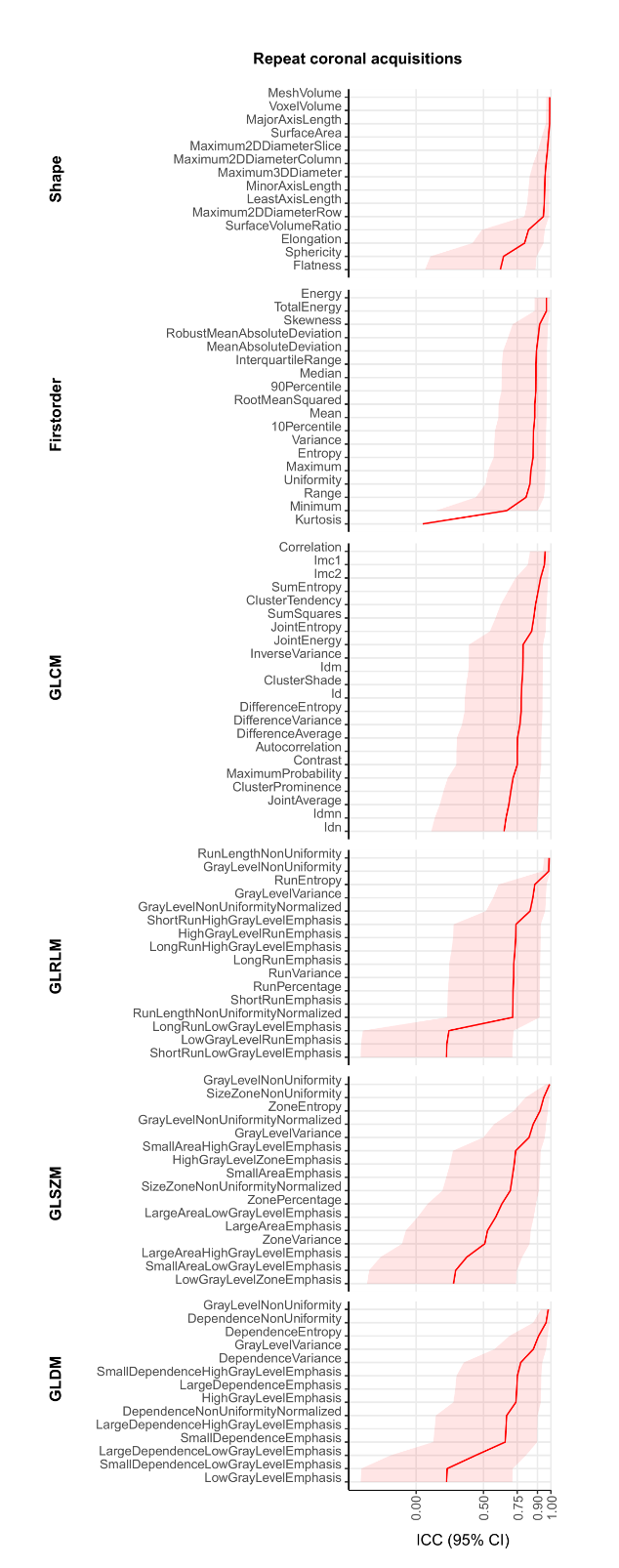


Supplementary figure 2


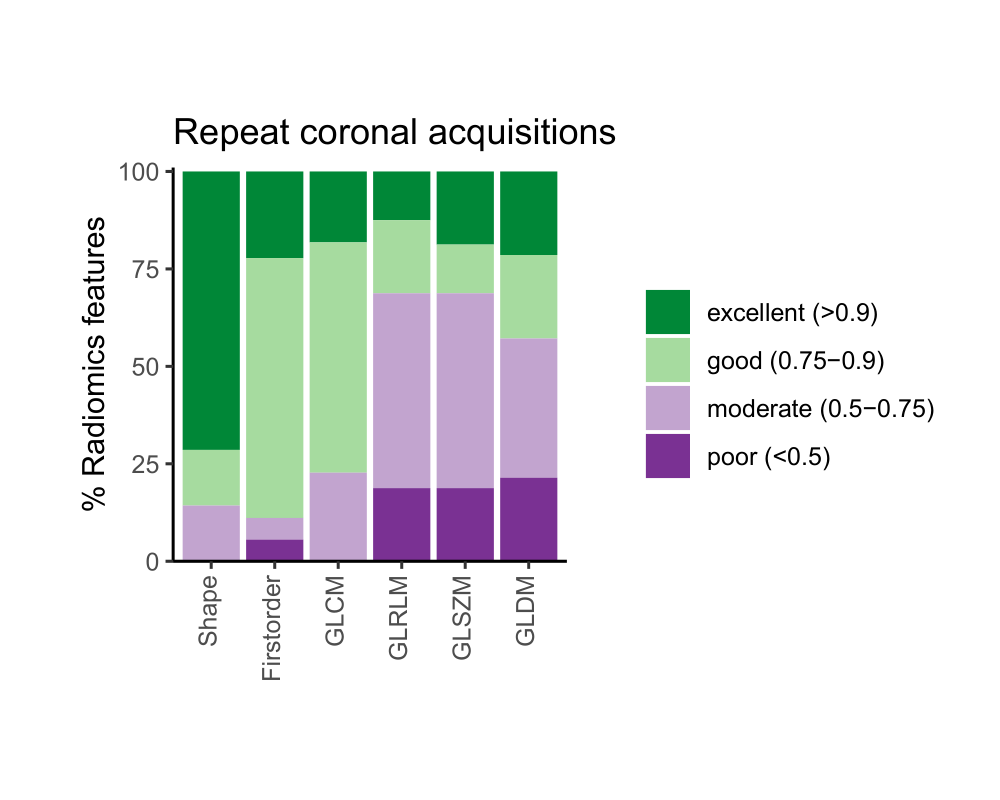

Supplement: Supplementary file 1 — (DOCX 3663 kb) [file 330_2022_9367_MOESM1_ESM.docx]
